# Development and Evaluation of *Glycine max* Germplasm Lines with Quantitative Resistance to *Sclerotinia sclerotiorum*

**DOI:** 10.3389/fpls.2017.01495

**Published:** 2017-08-31

**Authors:** Megan McCaghey, Jaime Willbur, Ashish Ranjan, Craig R. Grau, Scott Chapman, Brian Diers, Carol Groves, Mehdi Kabbage, Damon L. Smith

**Affiliations:** ^1^Department of Plant Pathology, University of Wisconsin-Madison, Madison WI, United States; ^2^Department of Crop Sciences, University of Illinois Urbana–Champaign, Champaign IL, United States

**Keywords:** *Glycine max*, *Sclerotinia sclerotiorum*, *Sclerotinia* stem rot, breeding, disease resistance, QTL

## Abstract

*Sclerotinia sclerotiorum*, the causal agent of Sclerotinia stem rot, is a devastating fungal pathogen of soybean that can cause significant yield losses to growers when environmental conditions are favorable for the disease. The development of resistant varieties has proven difficult. However, poor resistance in commercial cultivars can be improved through additional breeding efforts and understanding the genetic basis of resistance. The objective of this project was to develop soybean germplasm lines that have a high level of Sclerotinia stem rot resistance to be used directly as cultivars or in breeding programs as a source of improved Sclerotinia stem rot resistance. Sclerotinia stem rot-resistant soybean germplasm was developed by crossing two sources of resistance, W04-1002 and AxN-1-55, with lines exhibiting resistance to *Heterodera glycines* and *Cadophora gregata* in addition to favorable agronomic traits. Following greenhouse evaluations of 1,076 inbred lines derived from these crosses, 31 lines were evaluated for resistance in field tests during the 2014 field season. Subsequently, 11 Sclerotinia stem rot resistant breeding lines were moved forward for field evaluation in 2015, and seven elite breeding lines were selected and evaluated in the 2016 field season. To better understand resistance mechanisms, a marker analysis was conducted to identify quantitative trait loci linked to resistance. Thirteen markers associated with Sclerotinia stem rot resistance were identified on chromosomes 15, 16, 17, 18, and 19. Our markers confirm previously reported chromosomal regions associated with Sclerotinia stem rot resistance as well as a novel region of chromosome 16. The seven elite germplasm lines were also re-evaluated within a greenhouse setting using a cut petiole technique with multiple *S. sclerotiorum* isolates to test the durability of physiological resistance of the lines in a controlled environment. This work presents a novel and comprehensive classical breeding method for selecting lines with physiological resistance to Sclerotinia stem rot and a range of agronomic traits. In these studies, we identify four germplasm lines; 91–38, 51–23, SSR51–70, and 52–82B exhibiting a high level of Sclerotinia stem rot resistance combined with desirable agronomic traits, including high protein and oil contents. The germplasm identified in this study will serve as a valuable source of physiological resistance to Sclerotinia stem rot that could be improved through further breeding to generate high-yielding commercial soybean cultivars.

## Introduction

Soybean [*Glycine max* (L.) Merr.] is an important, globally grown source of protein, and it is the largest source of edible oil. In 2015, United States agricultural exports of soybean, soybean meal, and soybean oil had a value of nearly 28 billion dollars (USDA, 2016). In that year, soybean yielded an average of 3,195 kg ha^-1^ in the United States (National Agricultural Statistics Services, 2014–2016), which was a historical high. In 2016 in Wisconsin, seed oil concentration averaged 19.2% and protein averaged 34.3% (Miller-Garvin and Naeve, 2016), while food-grade soybean averaged 18.7% oil and 35.9% protein (Miller-Garvin and Naeve, 2016).

Among the factors limiting soybean production in the Midwestern United States is infection by *Sclerotinia sclerotiorum*, the causal agent of Sclerotinia stem rot (Sclerotinia stem rot). *S. sclerotiorum* is a destructive fungal pathogen in soybean and is estimated to have reduced yield by 1,606 million kilograms in 2009 (Koenning and Wrather, 2010). In other soybean growing regions such as Brazil, Sclerotinia stem rot has also become a production-limiting disease of soybean that can cause yield reductions as high as 60% (Cunha et al., 2010). Integrated management of Sclerotinia stem rot utilizes a combination of cultural, chemical, and biological control practices. Cultural practices include crop rotation, tillage, weed control, irrigation management, and modification of seeding rates and row spacing (Peltier et al., 2012). Fungicides such as picoxystrobin (Aproach^®^) and boscalid (Endura^®^) have resulted in suppression of Sclerotinia stem rot in field trials and are most effective when applied at the R1 (first flower) to R3 (beginning pod development) growth stages (Smith et al., 2014). The most commonly available and well-studied biological control agent for Sclerotinia stem rot is *Coniothyrium minitans* (Contans^®^) (Peltier et al., 2012). This beneficial fungus is known to degrade sclerotia, the resting structure of *S. sclerotiorum*.

Despite the existence of various tools for Sclerotinia stem rot management, a high level of control that does not rely on pesticide applications is still in dire need. Acceptable Sclerotinia stem rot control is limited by the lack of strong resistance in available commercial cultivars. Several partially resistant soybean genotypes have been identified in controlled environmental studies and field trials (Grau et al., 1982; Boland and Hall, 1987; Kim and Diers, 2000; Han et al., 2008; Huynh et al., 2010; Li et al., 2010; Sebastian et al., 2010; Bastien et al., 2014; Iquira et al., 2015; Zhao et al., 2015). Within partially resistant cultivars, various quantitative trait loci (QTL) contributing to Sclerotinia stem rot resistance have been identified. For example, three QTL were identified by Kim and Diers (2000) and 28 QTL were identified by Arahana et al. (2001) which individually explain 4–10% of the phenotypic variation for the trait. Additionally, Vuong et al. (2008) mapped four QTL for Sclerotinia stem rot resistance that each explained from 5.5 to 12.1% of the phenotypic variance in Sclerotinia stem rot development, and Guo et al. (2008) identified seven QTLs which explained 6.0–15.7% of resistance phenotype differences in their populations. Other studies of genetic resistance include investigations of the degradation of the *S. sclerotiorum* pathogenicity factor, oxalic acid, which resulted in the successful development of Sclerotinia stem rot resistant transgenic soybean (Donaldson et al., 2001; Cunha et al., 2010); and more recently the identification that the silencing of soybean NADPH oxidases leads to enhanced resistance to this pathogen (Ranjan et al., 2017). However, these transgenic soybeans have yet to be exploited commercially. Furthermore, a need persists to differentiate between structurally and physiologically resistant phenotypes, which are often not clearly distinguished in breeding lines.

Breeding for Sclerotinia stem rot resistance is complicated by polygenic resistance alleles, with some likely controlling structural disease avoidance phenotypes, such as plant height, and others controlling physiological resistance mechanisms, as well as complex genetic and environmental interactions. For example, Kim and Diers (2000) identified three QTL which accounted for 8–10% of disease severity index (DSI) variability. However, two were associated with disease klendusity (i.e., plant escape mechanisms) including plant height, lodging, and date of flowering. To determine physiological resistance to Sclerotinia stem rot, QTL have been mapped in greenhouse experiments where plants were inoculated to avoid screening for escape mechanisms associated with field trials (Arahana et al., 2001; Guo et al., 2008; Vuong et al., 2008). Physiological Sclerotinia stem rot resistance has, thus far, been limited to only a few partially resistant lines (Grau et al., 1982; Kim and Diers, 2000; Vuong et al., 2008). Field testing for physiological resistance is difficult, as environmental conditions and inoculum distributions are not uniform in field trials; the resulting differential disease pressure makes line comparisons unreliable.

Furthermore, isolates of *S. sclerotiorum* have been found to differ in aggressiveness. Willbur et al. (2017) highlighted the importance of using a representative panel of mildly to strongly aggressive isolates for screening soybean lines due to disparate interactions between isolates and lines which may be attributable to varying abilities of isolates to overcome host resistance mechanisms on certain genotypes. Additional efforts are needed to evaluate physiological resistance to ascertain related QTL and to breed for resistance to a wide range of isolates and environments.

Incomplete resistance in commercial soybean cultivars can be addressed through traditional breeding efforts and improved understanding of genetic sources of resistance while preserving the agronomic and industrial qualities of soybean. Breeding efforts have primarily focused on increasing yield first, before attempting to incorporate disease resistance traits. Furthermore, trade-offs can be expected when breeding exclusively for disease resistance due to associated energy requirements that may limit yield and metabolic activities (Wang et al., 2015). For example, lower lignin content of soybean is associated with disease resistance to Sclerotinia stem rot (Peltier et al., 2009). Lignin content, as a structural component of stems, may be inversely related to lodging which is a contributor to lower yields (Board, 2001). Furthermore, trade-offs have been observed historically when attempting to improve multiple traits simultaneously, which further complicates breeding efforts (Recker et al., 2014). Therefore, continuous evaluations of desirable traits are necessary for the development of elite soybean breeding lines.

The objectives of this project were to: (1) develop soybean germplasm lines that have a high level of Sclerotinia stem rot resistance, that yield competitively with commercial cultivars, while maintaining acceptable protein and oil profiles which would allow them to be used directly as cultivars or in breeding programs as a source of Sclerotinia stem rot resistance; (2) conduct a search for genetic markers associated with Sclerotinia stem rot resistance in the newly developed germplasm lines which can be used to select for resistance and to improve progress in breeding for Sclerotinia stem rot resistance; and (3) compare the response of the generated germplasm lines in a controlled greenhouse environment to multiple isolates of *S. sclerotiorum* and in field environments.

## Materials and Methods

### Breeding Line Generation

Six soybean populations were developed, utilizing the Sclerotinia stem rot resistance sources AxN-1-55 and W04-1002 (**Table 1**). AxN-1-55 (PI 640911) was released as public germplasm (Diers et al., 2006) with partial Sclerotinia stem rot resistance in 2006. AxN-1-55 has 75% plant survival after challenge with *S. sclerotiorum* in controlled inoculation trials (Grau *unpublished data*). W04-1002 is an inbred line derived from a single plant selection from PI 567157A (Peltier and Grau, 2008). W04-1002 has expressed 90 to 100% survival after repeated challenges with multiple isolates of *S. sclerotiorum* and is considered highly resistant to the pathogen (Peltier and Grau, 2008).

**Table 1:** Parental lines of initial crosses, desirable characteristics, plant introduction number, and line evaluation references.

Parental lines	Characteristics	Plant introduction^a^	Reference
W04–1002	Resistance to *S. sclerotiorum*	PI 567157A^b^	Peltier and Grau, 2008
AxN-1–55	Resistance to *S. sclerotiorum*	PI 640911	Diers et al., 2006
W04–571	High yield	Dwight x	Bachman et al., 1997
	Resistance to *C. gregata*	PI 567479^b^	Nickell et al., 1998
	Resistance to *H. glycines*		
W04–680	High yield	Dwight x	Bachman et al., 1997
	Resistance to *C. gregata*	PI 567479^b^	Nickell et al., 1998
	Resistance to *H. glycines*		
L84–5873	Resistance to *C. gregata*	PI 557536	Nickell and Bernard, 1992
LN89–5717	Resistance to *C. gregata*	PI 574542^b^	Hughes et al., 2004
	Resistance to HG type 6 of *H. glycines*		Nickell et al., 1994
Dwight^c^	High yield		Nickell et al., 1998
	Resistance to *H. glycines*		
	Sclerotinia stem rot susceptible control		

^a^*Plant introduction (PI) refers to the identifying number assigned to accessions within the National Plant Germplasm System. ^b^These lines are not regarded as ancestral lines for United States soybean cultivars and provided the potential for increased genetic diversity (Wang et al., 2005). ^c^Dwight was not a parental line of the populations in this paper, but it is present in pedigrees and was used as a susceptible control for Sclerotinia stem rot resistance assessments*.

The two aforementioned sources of resistance to *S. sclerotiorum* were selected as parents to cross with four parental lines possessing desirable agronomic and pathogen resistance traits (**Table 1**). Populations were assigned a name containing a number indicating the female parent and a “1” or “2” for the Sclerotinia stem rot resistant parent, W04-1002 or AxN-1-55, respectively. Population designations are as follows: 41 = W04-571 × W04-1002, 51 = W04-680 × W04-1002, 81 = L84-5873 × W04-1002, 91 = LN89–5717 × W04-1002, 42 = W04-571 × AxN-1-55, 52 = W04-680 × AxN-1-55.

Initial selections were made based on pod set, minimal lodging, maturity (MG0 to MGII), and absence of foliar diseases to ensure the persistence of acceptable agronomic qualities and parental *C. gregata* and *H. glycines* resistance. Six F1 seeds from each of the six populations were planted in a greenhouse; F2 seed was harvested and combined for field selection. In 2007 F2 seed was planted in a field nursery, naturally infested with *C. gregata* and *H. glycines*, at West Madison Agricultural Research Station located in Verona, Wisconsin (43.06028, -89.531667). Approximately 300 plants were selected within each population, harvested, and F3 seed was combined for planting in 2008. In 2008 field selections, the identity of progeny of each selected plant was maintained to develop sets of individual F3:4 breeding lines for each population.

Selection for resistance to *S. sclerotiorum* was conducted in greenhouse studies and in naturally infested field nurseries. Four plants per F3:4 line were challenged with *S. sclerotiorum* isolate 105HT (Peltier and Grau, 2008) in greenhouse trials to select for physiological resistance. A cut-petiole inoculation technique was used to challenge lines at the R1 (first flower) growth stage (Peltier and Grau, 2008). One or two surviving plants were advanced to the next generation of selection and identified as a new line. Remnant seed from lines designated as susceptible (plants from lines with 100% mortality when inoculated) were planted to maintain both resistant and susceptible lines within each population in order to assess genetic gain from selection. Single plant selection of new lines continued until the F7 generation, and 1,076 F7:8 lines were advanced to the field for further selection.

After three generations of greenhouse selection, 1,076 inbred lines (F7:8) were planted in 6.1 m, single row, non-replicated plots in a field nursery naturally infested with *C. gregata* and *H. glycines*. Eight hundred and thirty lines were selected for the persistence of acceptable agronomic traits and disease resistance based on criteria previously described. All breeding lines (F7:9 generation) within the four W04-1002-descended populations were once again evaluated for resistance to *S. sclerotiorum* in greenhouse trials. Selection within population 42 was discontinued due to a lack of sustained and measurable disease resistance. By the end of this selection phase of the project, there were 109 lines for population 41, 117 lines for population 51, 224 lines for population 81 and 250 lines for population 91 for a total of 700 lines.

### 2013 Preliminary Greenhouse Disease Severity Evaluations

Greenhouse trials were conducted in 2013 at the West Madison Greenhouse Complex located on the grounds of West Madison Agricultural Research Station. Soybean seeds were planted approximately 4 cm deep in 15.25 cm diameter pots of moist potting mix (Sun Gro Horticulture). Soybean plants were watered daily and fertilized twice weekly (Scotts Peters Professional Peat-Lite Special 20-10-20; Scotts-Sierra Horticultural Products Co.) prior to inoculation.

Soybean plants were inoculated using the cut petiole technique (Peltier and Grau, 2008) with aggressive *S. sclerotiorum* isolate 25 (Willbur et al., 2017). A 1.5-cm-thick agar core was collected from the leading edge of mycelia on each inoculum plate with a 1,000 μl pipet tip (Fisher Scientific). At the R1 (first flower) growth stage, second or third trifoliate leaflets were excised at a petiole length of 2.5 to 3 cm. Pipet tips of inoculum were placed on petioles such that mycelia and cut petiole tissue were in direct contact. Two to three plants (sub-samples) were inoculated per pot for each line and replicated three to four times in a randomized complete block design (RCBD) blocked by replicate. The trial was repeated once. F7:9 lines were phenotyped at the R3 growth stage 14 days post-inoculation (DPI) for resistance to Sclerotinia stem rot using a rating scale of 0 (no stem lesion), 1 (small stem lesion), 2 (lesion but no wilt), 3 (wilt), and 4 (dead plant). Lines with a mean severity score of 0 to 1 were characterized as resistant.

### Genetic Marker Analysis

This work focused on mapping genes that control Sclerotinia stem rot infection using the W04-1002 lines as a resistance source because it represents a novel and stable source of Sclerotinia stem rot resistance. Genomic DNA was extracted from a bulk sample of fresh leaf tissue from each of the 8–10 most Sclerotinia stem rot-resistant and most Sclerotinia stem rot-susceptible lines in 2013 greenhouse evaluations (**Table 2**). Seven soybean leaves for each line were used for the hexadecyltrimethylammonium bromide (CTAB) extraction protocol as described by Keim et al. (1988). The samples were tested for single nucleotide polymorphism (SNP) genetic markers with the Illumina GoldenGate 1,536 Universal Soy Linkage Panel 1.0 (USLP 1.0) (Hyten et al., 2010). Marker data were analyzed for an association between disease resistance and the alleles from the resistant and susceptible parents for each marker. Based on chi-square analysis, SNP markers with significant segregation distortion were identified. Subsequently, microsatellite markers closely linked to the significant SNP markers from the Chi-square analysis were used to evaluate all lines in the populations. Primer sequences for microsatellite markers were obtained from the SoyBase website^1^. Genetic markers were evaluated for 109 lines in population 41, 117 lines in population 51, 224 lines in population 81 and 250 lines in population 91, for a total of 700 lines evaluated. Lines were tested with 12 to 37 markers, depending on the population, that mapped onto three to seven chromosomes (**Table 3**).

**Table 2:** Number of most resistant and susceptible lines in each population from 2013 Sclerotinia stem rot greenhouse evaluations used for performing SNP marker analysis.

Population (Parents)	Resistant	Susceptible
4x1 (LW04–571 × W04–1002)	9	9
5x1 (LW04–680 × W04–1002)	8	8
8x1 (L84–5873 × W04–1002)	10	10
9x1 (LN89–5717 × W04–1002)	8	7

**Table 3:** The parents of the populations tested for resistance and genetic markers, the number of lines in each population, the number of markers used to test the populations, and the chromosomes where markers are located.

Population	Parents	Number of lines	Number of markers	Chromosomes
4 × 1	LW04–571 × W04–1002	109	25	2, 3, 4, 8, 10, 15, 19
5 × 1	LW04–680 × W04–1002	117	37	2, 8, 9, 16, 18, 19
8 × 1	L84–5873 × W04–1002	224	12	15, 17, 18
9 × 1	LN89–5717 × W04–1002	250	25	6, 10, 12, 16, 19

### Field Evaluations of Agronomic Traits and Disease Severity of Later Generation Breeding Lines

Lines planted in the 2014 advanced field trials were selected based on the lowest Sclerotinia stem rot disease severity of the 336 lines trialed from the six populations in the 2013 naturally infested field trials (population 41 = 64 lines; population 51 = 51 lines; population 81 = 93 lines; population 91 = 76 lines; population 42 = 36 lines; population 52 = 16 lines). Subsequent evaluations were also performed in the greenhouse in early 2014. As mentioned previously, population 42 was not evaluated through 2015 due to a lack of performance.

The lines selected in 2013 were planted at the West Madison Agricultural Research Station in May of 2014 and the Hancock Agricultural Research Station in May of 2015 and 2016. Plots were overhead irrigated at each location 1.9–3.2 cm/ha every 2–5 days to facilitate disease development.

The experimental design each year was a randomized complete block (blocked by replicate) with five or six replications. Plots consisted of four 0.76 m wide rows that were 6.1 m long. Each plot was separated by a 1.5 m non-planted alley. Sowing occurred at a rate of approximately 437,500 seeds ha^-1^ using a tractor-mounted cone-type planter (Almaco, Nevada, IA). Nutrient management was conducted per University of Wisconsin-Madison Cropping Guidelines.

Plot grain weight and moisture was taken from the two center rows of each field plot using an Almaco (Nevada, IA, United States) SPC40 small-plot combine equipped with a HarvestMaster HM800 grain gauge with Mirus software package (Juniper Systems, Logan, UT, United States). Yield measurements were calculated and standardized to 13% moisture. Sub-samples of grain were obtained during harvest and used to assess oil (%) and protein (%) in 2015 and 2016. Oil and protein data were assessed using the average of five 50 ml subsamples of seed from each plot using a near infrared (NIR) grain analyzer (Perten Instruments Inframatic 9500, Hägersten, Sweden). Readings were calibrated by the system for a moisture content of 13%.

Sclerotinia stem rot severity index (DSI) was determined in all years by a rating 30 arbitrarily selected plants in each plot of the field nursery at the R6 soybean growth stage. Plants were scored either 0 (no infection), 1 (infection on branches), 2 (infection on, but not girdling, the main stem), or 3 (infection on the main stem resulting in death or poor pod fill). The sum of the scores of the 30 plants were totaled for each class and divided by 0.9 (Grau et al., 1982). The disease incidence (DI) was calculated by counting the number of symptomatic plants in 12.19 m of row. Lodging was measured October 11, 2014, October 10, 2015, and October 14, 2016 using an average ranking for each plot of 1 (no leaning), 2 (25-degree lean), 3 (45-degree lean), 4 (more than a 45-degree lean), and 5 (laying on the ground) for each plot.

### Late Generation Multi-Isolate Greenhouse Evaluations of AUDPC

Multi-isolate greenhouse evaluations were conducted on soybean plants from the seed of lines tested in 2016 using the previously described cut-petiole technique (Peltier and Grau, 2008). Three soybean seeds per pot were planted approximately 4 cm deep in 15.25 cm diameter peat pots of moist potting mix (Premier Pro-Mix HP BioFungicide + Mycorrhizae). Nine *S. sclerotiorum* isolates, of 44 previously characterized isolates with varying degrees of aggressiveness (Willbur et al., 2017), were used for evaluations. As described by Willbur et al. (2017), lines were inoculated at the V4 growth stage, and lesions were measured with digital calipers (Thermo Fisher Scientific) 5, 12, and 14 DPI. Inoculations were performed in triplicate (three replicates) with three seeds planted per pot, and soybean plants were arranged in a RCBD blocked by replicate. Line evaluations were repeated a second time. Initial inoculum was generated from dry-stored sclerotia (Willbur et al., 2017). Inoculum applied in the second repetition was generated from sclerotia reisolated from the first repetition of plants. The AUDPC was analyzed to evaluate germplasm resistance reactions to a variety of isolates in both greenhouse screens.

### Statistical Analysis

Mixed-model analysis of variance (ANOVA) was conducted for lodging, agronomic traits, and protein and oil using PROC GLIMMIX in the SAS statistical software package and the analysis of the markers tested for populations was conducted in PROC GLM (v 9.4, SAS Institute, Inc. Cary, NC, United States). Means were separated using Fisher’s Least Significant Difference (LSD) via an open source macro (Piepho, 2012). Prior to analysis, lodging scores for each plot were subjected to rank analysis using PROC RANK in SAS. This was done to normalize the categorical nature of lodging scores, so that mixed model ANOVA could be conducted as described above. Disease and yield data were analyzed separately for each year due to large differences in overall disease attributable to environment variability. Significance was reported at α = 0.05 significance level.

The multi-isolate-germplasm line experiments were analyzed using a generalized mixed model (PROC GLIMMIX) analysis of variance using SAS (v 9.4, SAS Institute, Inc.), as described in Willbur et al. (2017). Data were normalized using a lognormal distribution and denominator degrees of freedom for fixed effects were computed using the Kenward-Rodger degrees of freedom approximation. Differences between lines and isolates were determined at α = 0.05 significance level.

## Results

### Germplasm Generation, and 2013 Greenhouse Disease Severity Evaluations

**FIGURE 1:**
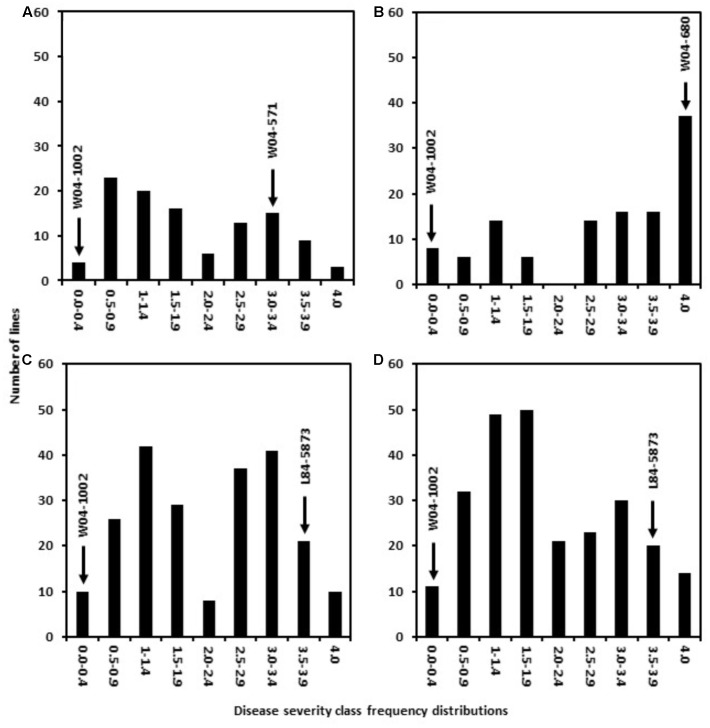
Frequency distribution of disease severity classes in populations 41 **(A)**, 51 **(B)**, 81 **(C)**, and 91 **(D)** from 2013 greenhouse evaluations. The resistant parent line W04–1002 showed a highly resistant rating of 0.1 while the average rating of other parental lines was 3.0 for W04–571, 4.0 for W04–680, 3.5 for L84–5873, and 3.8 for LN89–5717.

Six populations were generated in this study by utilizing two Sclerotinia stem rot resistance sources AxN-1-55 and W04-1002. Crosses were established between these sources of resistance and six parental lines conferring other desirable pathogen resistance traits (See materials and methods for details). The resulting populations were designated as follows: 41 = W04-571 × W04-1002, 51 = W04-680 × W04-1002, 81 = L84–5873 × W04-1002, 91 = LN89–5717 × W04-1002, 42 = W04-571 × AxN-1-55, 52 = W04-680 × AxN-1-55. In 2013, 700 promising inbred lines (F7:9) derived from W04-1002, were subjected to *S. sclerotiorum* petiole inoculations to evaluate physiological resistance to *S. sclerotiorum* and to later identify markers associated with resistance phenotypes. Responses observed among the lines ranged from resistant to highly susceptible within greenhouse trials (**Figure 1**). After multiple greenhouse trials, 160 of 700 expressed 0 to 25% plant mortality (severity class 0–1, **Figure 1**). Concurrently, parental lines were also evaluated for Sclerotinia stem rot resistance. As expected, the resistant parent W04-1002 showed a highly resistant rating of 0.1, while the average rating of W04-571 was 3.0, W04-680 was 4.0, L84–5873 was 3.5, and LN89–5717 was 3.8. W04-1002, therefore, remained one of the most resistant lines, and a range of responses to Sclerotinia stem rot persisted among lines at the F7:9 generation. The results of 2013 greenhouse evaluations informed selection of the most resistant and susceptible lines for SNP analyses in 2013 and the selection W04-1002 lines evaluated in 2014 field trials.

### Genetic Markers Associated with Sclerotinia Stem Rot Resistance

The preliminary marker analysis was performed using 1,536 SNP genetic markers (data not shown). This was done by comparing the marker pattern of the 8–10 most resistant lines with the 8–10 most susceptible lines in each population generated from a cross with W04-1002 (**Table 2**). The SNP markers data were analyzed to determine if there was an association between disease resistance and the alleles from the resistant and susceptible parents for each marker. This association was determined by testing for significant segregation distortion compared to expected random segregation. A significant distortion of segregation indicated that the marker was genetically close to a resistance allele. Based on chi-square analysis, markers were identified that had significant (*P* < 0.05) segregation distortion. Due to the high cost of testing all lines in the four populations with the 1,536 SNP markers, microsatellite markers in regions where the distorted markers are located were then used to test all lines in the populations (**Table 3**).

**FIGURE 2:**
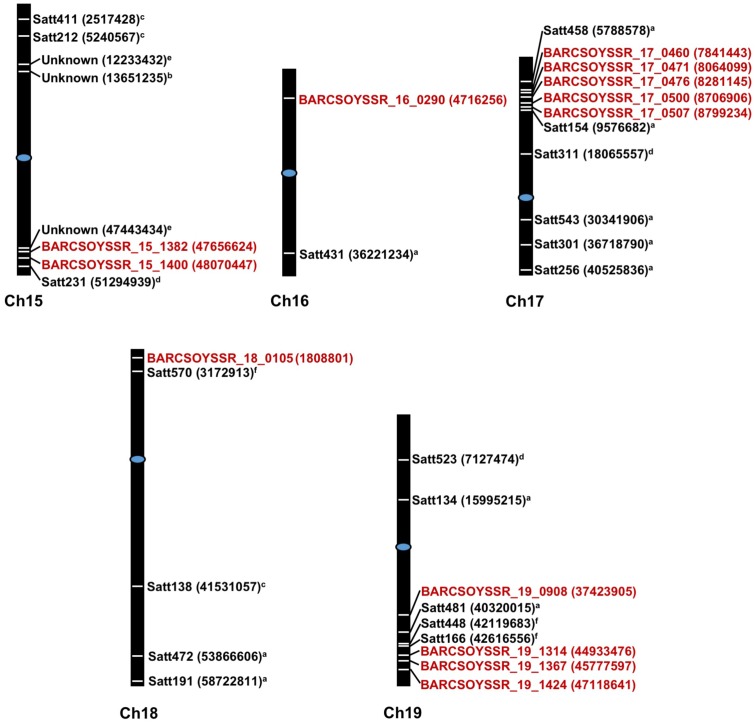
Physical starting positions of markers associated with disease resistance in previous studies and this study. Chromosome numbers are displayed under each chromosome. Markers locations are indicated by white lines placed directly on chromosomes and are placed relative to physical positions. Physical starting position of the microsatellite markers are shown in parenthesis. Markers in red text are from the present study while markers from previous study are shown in black text. Centromeres are indicated by blue ovals. ^a^Arahana et al., 2001; ^b^Bastien et al., 2014; ^c^Guo et al., 2008; ^d^Han et al., 2008; ^e^Iquira et al., 2015; ^f^Sebastian et al., 2010.

The microsatellite marker results from all lines in the populations were then combined with the resistance data from the 2013 greenhouse evaluations to map QTL in each population. Using a significance threshold of *P* < 0.05, this analysis resulted in the mapping of QTL to one region on chromosome 15 in population 41, one region on chromosome 19 in population 51, regions on chromosomes 17 and 18 in population 81, and regions on chromosomes 16 and 19 in population 91 (**Table 4**). For many of these regions, multiple significant markers were identified in each population, but these areas where multiple markers arise are likely linked to a common resistance QTL (**Figure 2**). Additionally, we mapped the physical location of our significant markers with previously published markers associated with Sclerotinia stem rot resistance. With the exception of a novel position on chromosome 16, the majority of our markers confirmed previously identified genetic hot spots associated with Sclerotinia stem rot resistance (**Figure 2**).

**Table 4:** Significant genetic markers identified in the analysis of the four populations evaluated for Sclerotinia stem rot severity in 2013 greenhouse evaluations.

Marker	Chromosome number	*P*-value	*S*^a^	*R*^b^	Chromosome position (bp)^c^
**41 Population**					
BARCSOYSSR_15_1382	15	0.05	2.2	1.7	47,656,624
BARCSOYSSR_15_1400	15	<0.01	1.6	2.2	48,070,447
**51 Population**					
BARCSOYSSR_19_1314	19	0.04	3.1	2.7	44,933,476
BARCSOYSSR_19_1367	19	<0.01	3.1	2.7	45,777,597
BARCSOYSSR_19_1424	19	0.04	3.1	2.7	47,118,641
**81 Population**					
BARCSOYSSR_17_0460	17	<0.01	2.5	2.0	7,841,443
BARCSOYSSR_17_0471	17	<0.01	2.5	2.1	8,064,099
BARCSOYSSR_17_0476	17	0.05	2.5	2.1	8,281,145
BARCSOYSSR_17_0500	17	0.04	2.4	2.1	8,706,906
BARCSOYSSR_17_0507	17	0.02	2.4	2.1	8,799,234
BARCSOYSSR_18_0105	18	0.02	2.1	2.5	1,808,801
**91 Population**					
BARCSOYSSR_16_0290	16	0.04	2.2	1.9	4,716,256
BARCSOYSSR_19_0908	19	0.05	2.2	1.9	37,423,905

^a^*S column contains the disease severity phenotypic means of the lines that are homozygous for the marker allele from the susceptible parent. ^b^*R* column contains the disease severity phenotypic means of lines that are homozygous for the marker allele from the Sclerotinia stem rot resistant parent of the populations (W04–1002). ^c^The chromosome positions for Sclerotinia stem rot markers are from the Glyma.Wm82.a2 assembly at soybase.org*.

### Field Evaluations of Agronomic Traits and Disease Severity of Late Generation Breeding Lines

After greenhouse evaluations, 31 lines including parental lines and the susceptible controls, Dwight and 91–44, were evaluated for Sclerotinia stem rot severity and the important agronomic traits of yield and lodging. Line performance in-field provided an assessment of the use and commercialization potential of lines. Significant differences among lines were observed in 2014 field tests for Sclerotinia stem rot severity (*P* < 0.0002) and incidence (*P* < 0.0001) (**Table 5**). The experimental line most susceptible to Sclerotinia stem rot based on DSI and DI in the 2014 field test was 91–44 which had a higher DSI and DI than all other lines. Lines SSR81–107, SSR51–70, and the parental lines had DSI and DI ratings significantly lower than 91–44 but not different from most lines (**Table 5**). AxN-1-55 yielded higher than all cultivars and lines, however, Dwight, 52–11, and 52–82B were not significantly different from this line. Therefore, all breeding lines were significantly lower in DSI and DI than the susceptible line, 91–44, and lines from population 52 had promising yields.

**Table 5:** Sclerotinia stem rot severity and incidence, and yield of soybean breeding lines and cultivars tested in a Sclerotinia stem rot nursery at University of Wisconsin’s Hancock Agricultural Research Station, 2014–2016.

Breeding line or cultivar	Sclerotinia stem rot DSI (0–100)^a,c^			Disease Incidence (# of plants)^b,c^			Yield (kg ha^-1^)^c^		
	2014	2015	2016	2014	2015	2016	2014	2015	2016
91–44	59.1 a	50.2 a	85.2 ab	36.8 a	32.0 cd	34.9 ab	2,215.9 gh	3,058.2 cde	2617.0 c
52–14	22.7 b	15.6 bd	–	9.8 b	10.0 bc	–	2,915.5 bcd	3,541.6 ac	–
41–39	22.2 b	6.6 bc	–	9.2 bc	3.5 cd	–	2,495.0 efg	2,932.6 de	–
51–23	17.1 bc	6.9 bc	59.7 cd	8.4 bc	3.0 a	21.6 bcd	2,625.6 df	3,156.2 bcd	3135.6 b
91–103	16.2 bc	4.7 cd	–	7.2 bc	2.8 cd	–	2,355.0 fg	2,662.2 df	–
Dwight	15.3 bc	50.9 a	91.8 a	8.6 bc	26.6 cd	41.9 a	3,102.0 ab	3,486.5 ac	2621.0 c
52–11	14.0 bc	11.8 bc	72.0 ac	10.0 b	5.2 cd	33.0 ab	3,232.6 ab	3,134.3 cd	3522.8 ab
52–82B	13.1 bc	4.0 cd	65.4 bc	5.6 bc	1.4 c	28.7 abc	3,075.7 ac	3,742.8 a	3822.9 a
91–145	10.9 bc	5.4 bc	56.2 cd	3.8 bc	2.4 a	15.8 d	2,336.6 fh	2,246.5 f	3459.9 ab
91–38	9.3 bc	10.7 bc	38.9 de	3.2 bc	4.8 cd	14.2 de	2,456.4 efg	2,605.5 ef	3345.6 b
SSR81–62	8.4 bc	14.6 bc	–	4.8 bc	8.4 bd	–	2,753.6 cde	2,880.3 de	–
SSR51–70	5.6 c	2.9 cd	23.5 ef	1.8 bc	1.2 d	8.7 e	2,252.5 gh	2,801.8 de	3187.7 b
AxN-1–55	3.8 c	7.8 bc	–	1.2 bc	3.6 cd	17.8 cd	3,356.9 a	3,667.8 ab	3865.2 a
W04–1002	3.8 c	2.9 c	16.5 f	1.6 bc	1.0 cd	4.1 f	1,994.7 h	2,258.3 f	3073.8 bc
SSR81–107	2.0 c	17.1 b	–	0.6 c	10.4 cd	–	2,226.5 gh	2,612.0 ef	–

^a^*Sclerotinia stem rot severity was measured using the disease severity index (DSI) which was generated by rating 30 arbitrarily selected plants in each plot and scoring plants on a 0–3 scale: 0, no infection; 1, infection on branches; 2, infection on main stem with little effect on pod fill; 3, infection on main stem resulting in death or poor pod fill. The scores of the 30 plants were totaled for each class and divided by 0.9. ^b^Average number of symptomatic plants in 12.19 m of row. ^c^Means followed by the same letter are not significantly different based on Fisher’s Least Significant Difference (LSD; α = 0.05)*.

Similarly, lines were evaluated for Sclerotinia stem rot and agronomic traits in 2015. Differences between disease responses were also present in 2015 (*P* < 0.0001) (**Table 5**). In 2015, the susceptible check, Dwight, and line 91–44 had significantly greater DSI scores than any other line, 50.9 and 50.2, respectively. Lines SSR51–70, W04–1002, 52–82B, and 91–103 had among the lowest DSI scores (<5.0). Dwight, 52–14, AxN-1–55, and 52–82B were the highest yielding of all lines (>3,480 kg ha^-1^), while the lowest yielding lines included the highly Sclerotinia stem rot-resistant lines W04–1002 and 91–145 (<2,300 kg ha^-1^). Breeding lines again demonstrated better resistance than susceptible lines, and lines such as 52–82B exhibited both the desirable phenotypes of low disease and high yield.

Among the 10 lines evaluated in 2016, significant differences were present among lines for DSI (*P* < 0.01) and DI (*P* < 0.01) (**Table 5**). DSI and DI values were much higher in 2016 compared to previous years. The most susceptible lines were Dwight, 91–44, and 52–11 with respective DSI values of 91.8, 85.2, and 72.0. In addition to these lines, 52–82B also had a higher DI compared to all but three of the lines. Lowest DSI rankings occurred in lines SSR51–70 and W04–1002 with DSI values of 23.5 and 16.5, respectively. 91–38 also exhibited low disease severity levels that were not significantly different from SSR51-70. Yield in 2016 was greater than in previous years, and the highest yields occurred in lines AxN-1–55 and 52–82B, 3,865.2 and 3,822.9 kg ha^-1^, respectively. Lines 91–145 and 52–11 yielded similarly to these lines. Lines with the lowest yields were Dwight and 91–44. Interestingly, results demonstrated high disease ranking for the high yielding varieties 52–11 and 52–82B and lower DSI for SSR51–70 compared to many lines, as previously observed. This outcome occurred despite unusually high disease levels in a field naturally inoculated with *S. sclerotiorum* infected sunflowers.

In 2016 field nurseries, 91–38 and AxN-1–55 had the least lodging (*P* < 0.05) with mean lodging scores that were not significantly different from high yielding lines, 52–11and 52–82B (Supplementary Table S2). Lines 91–145 and SSR51–70 exhibited the highest lodging scores, 3.4 and 3.2, respectively, and were not significantly different from 91 to 44, which had a score of 2.4. Differences in lodging ranks between lines were observed in 2014 (Supplementary Table S1) and 2016, but not 2015. 91–38 and 52–11 were not significantly different from AxN-1–55 in 2014 (Supplementary Table S1). Lodging results indicated the problematic trait of lodging was consistently present in the highly resistant line, SSR51–70. However, other lines such as 91–38 and 52–82B exhibited positive traits such as moderate disease resistance and high yield in conjunction with a good stand.

In 2015, protein and oil were added to the agronomic traits evaluated, as they are important considerations for commercialization and breeding (Supplementary Table S3). The selected breeding lines were also evaluated in 2016 (Supplementary Table S4). In 2016, the line with the highest protein content, 39.4%, was W04–1002 (*P* < 0.05), and it was not significantly different from SSR51–70 at 39.2 % and 91–44 at 38.7%. The highest percentage of oil, 19.2%, was measured from 91 to 44 and 91 to 38. Similarly, the aforementioned lines produced high levels of protein and oil in 2015 (Supplementary Table S2).

Overall, after 3 years of evaluations for disease responses and agronomic traits, genetic gain was observed within breeding populations. Desirable observed traits include high levels of disease resistance, as observed in SSR51–70, and maintained yields, as observed with 52–82B. Additionally, moderate disease resistance was observed in conjunction with high protein and oil or moderate yield as observed in 91–38 and 51–23, respectively. Therefore, field evaluations elucidated several promising lines for future breeding or commercialization.

### Late Generation Multi-Isolate Greenhouse Evaluations of AUDPC

**FIGURE 3:**
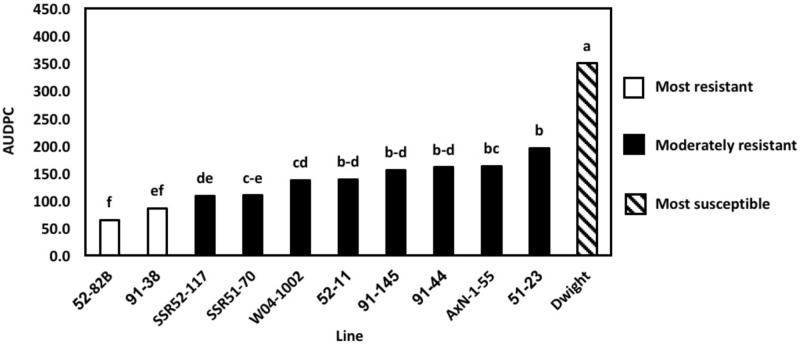
Area under the disease progress curve (AUDPC) of multi-isolate greenhouse evaluations. Means followed by the same letter are not significantly different based on Fisher’s Least Significant Difference (LSD; α = 0.05). LSD values were calculated based on the lognormal transformed AUDPC values.

To determine the physiological resistance of lines, in the absence of field escape mechanisms, greenhouse inoculations of the 2016 lines were conducted using nine previously characterized (Willbur et al., 2017) *S. sclerotiorum* isolates. Previously, 2013 greenhouse evaluations used only a single aggressive isolate. However, current *S. sclerotiorum* research indicates that various isolates may elicit differential resistance responses (Willbur et al., 2017). In 2016, differences in the AUDPC of lines, measured at 5, 10, and 14 DPI, were explained by the line inoculated (*P* < 0.01) and the isolate used (*P* < 0.01. The most resistant lines, as indicated by the lowest AUDPC, were 52–82B and 91–38 (*P* < 0.05) (**Figure 3**). AUDPC results were lower than the resistant parents,’ and these lines outperformed several lines considered more resistant based on results from field evaluations. For example, SSR51–70 was consistently more resistant in field experiments than 52–82B and 91–38. Genetic gain was once more observed, as 52–82B and 91–38 had lower AUDPCs than resistant parents. Additionally, 52–82B and 91–38 are promising lines for agronomic traits, as previously observed. Therefore, high levels of Sclerotinia stem rot resistance bolsters their applicability as commercial lines. Overall, 2016 multi-isolate greenhouse evaluations demonstrated the importance of pathogen diversity and screening in a controlled environment for physiological resistance and the broad applicability of breeding efforts.

## Discussion

In this study, QTL associated with reduced *S. sclerotiorum* infection were identified in the four populations of germplasm lines generated from W04–1002 as a source of Sclerotinia stem rot resistance. QTL were identified on chromosomes 15, 16, 17, 18, and 19. These QTL can be used in soybean breeding programs to facilitate the development of Sclerotinia stem rot resistant varieties through marker-assisted selection. For most the significant markers, the allele associated with a lower disease severity phenotype originates from W04–1002, the resistant parent of the populations reported here. However, for marker 1400 from population 41 and marker 0105 from population 81, the allele associated with lower disease severity originated from the susceptible parent (**Table 4**). Resistance alleles for QTL from susceptible parents have been previously identified in other studies (Toojinda et al., 1998). Associated phenotypes included a reduction in lesion size caused by *S. sclerotiorum* on soybean (Arahana et al., 2001) and resistance to BSR originating from PI88788, after crossing with another susceptible parent, potentially through epistatic interactions (Bachman and Nickell, 2000; Patzoldt et al., 2005). Epistatic interactions are corroborated by the findings of Moellers et al. (2017), which identified 24 significant epistatic interactions related to Sclerotinia stem rot resistance through genome-wide associated epistatic studies.

Previously, QTL conferring resistance to Sclerotinia stem rot have been reported for the chromosomes identified in this study. Based on genomic map searches on SoyBase.org and a review of current literature on Sclerotinia stem rot resistance loci, it is possible that some of the microsatellite markers in this study correspond to previously identified Sclerotinia stem rot resistance QTL (Boland and Hall, 1987; Kim and Diers, 2000; Arahana et al., 2001; Guo et al., 2008; Han et al., 2008; Vuong et al., 2008; Huynh et al., 2010; Li et al., 2010; Sebastian et al., 2010; Bastien et al., 2014; Zhao et al., 2015). On chromosomes 15, 17, 18, and 19 markers are located between 0.77 and 4.5 Mega base pairs (Mbp) from the closest, previously associated markers: BARCSOYSSR_17_0507 with Satt154 (Arahana et al., 2001) and BARCSOYSSR_19_1424 with SATT166 (Sebastian et al., 2010), respectively. However, it is likely that a marker identified on chromosome 16, BARCSOYSSR_16_0290 is associated with a novel source of resistance, as it is located 31.5 Mbp from the closest, previously identified marker, Satt431 (Arahana et al., 2001). Arahana et al. (2001) also identified chromosome 16 as an important contributor to Sclerotinia stem rot resistance. Conversely, they did not find a strong effect in a single population and only observed an association when populations were combined. In this report, we demonstrated that a single population, population 91, possesses a significant QTL on chromosome 16 (**Figure 2** and **Table 4**). It is important to note that Arahana et al. (2001) and Han et al. (2008) did find significant associations between resistance to Sclerotinia stem rot and markers on chromosome 16 for which the physical position is not available in the literature searched or SoyBase.org. This study both confirms the presence of QTL near regions identified in previous studies and presents a novel locus (BARCSOYSSR_16_0290), which may be useful in breeding for resistance to Sclerotinia stem rot.

These results are consistent with previous studies showing that Sclerotinia stem rot resistance QTL typically have small effects and are therefore difficult to map (Kim and Diers, 2000; Arahana et al., 2001; Vuong et al., 2008). An important consideration with the results presented here is that the resistance testing was done in a greenhouse with a reliable inoculation technique to directly assess physiological resistance. Previous efforts to map QTL associated with field resistance have instead resulted in the identification of markers associated with architectural traits such as plant height and lodging (Kim and Diers, 2000). Plant architecture should not have had a major impact on the resistance phenotypes observed in these greenhouse studies; the QTL identified in this study, therefore, are likely not associated with architectural traits. Additionally, QTL on chromosomes 15 and 18 were previously identified in association with cut-petiole assays conducted in the field (Guo et al., 2008); QTL identified on chromosomes 16, 17, and 19, however, were associated with Sclerotinia stem rot resistance in detached leaflet assays (Arahana et al., 2001). This study confirms that QTL on chromosomes 16, 17, and 19 are associated with Sclerotinia stem rot resistance and are, furthermore, associated with physiological resistance in whole-plant, cut-petiole inoculations which is likely more representative of a true resistance phenotype. Several lines in the populations presented here, possess QTL on chromosomes 16 and 19 that were identified using our techniques. Therefore, soybean breeders may find these QTL more useful than previously thought based on the strong response identified using our whole-plant inoculations and field screening.

The results from the multi-isolate greenhouse evaluations demonstrate the importance of selection within a controlled greenhouse environment for determining a high level of physiological resistance. Representative isolates caused a range of resistance reactions as previously described by Willbur et al. (2017) and variation in isolate aggressiveness has been reported previously (Kull et al., 2004; Li et al., 2008; Vleugels et al., 2013). These studies provide evidence that the breeding lines in this study have been confirmed to exhibit durable resistance to multiple *S. sclerotiorum* isolates, which substantiates the high level of partial resistance available in these populations.

Multi-isolate greenhouse evaluation results were consistent when repeated. However, results differed from field trials in some cases. Lines 52–82B and 91–38 exhibited the highest levels of resistance in greenhouse trials but not in field trials and SSR51–70 did not perform as well in greenhouse evaluations, indicating the importance of using controlled environment evaluations to elucidate physiological resistance phenotypes. The results of Willbur et al. (2017) corroborated moderate resistance in 91–38 against multiple *S. sclerotiorum* isolates in greenhouse evaluations. This suggests klendusity for pathogen avoidance in some germplasm lines, which become apparent in a field setting. Furthermore, partial resistance in some lines may be overcome in the field if cool, moist environments, adequate inoculum, and the correspondence of flowering with apothecial development are simultaneously met in years favorable for Sclerotinia stem rot. Marker alleles corresponding to higher DSI of Sclerotinia stem rot on soybean have also been identified in association with phenotypes of taller plant height, greater lodging, and later flowering (Kim et al., 1999). As a result, differences in disease severity in a field setting may be a direct effect of physical, rather than physiological, attributes that prevent favorable infection conditions. These studies suggest that a combination of greenhouse inoculations for elucidating physiological resistance and subsequent field evaluations for Sclerotinia stem rot field resistance and agronomic properties contribute to a holistic method to identify lines with QTL for Sclerotinia stem rot resistance and to comprehensively characterize resistance in breeding programs.

This work demonstrates that genetic gain can be made for Sclerotinia stem rot resistance in soybean while maintaining agronomic qualities, protein and oil content, and resistance to other pathogens. Breeding efforts using a novel source of Sclerotinia stem rot resistance followed by greenhouse and field screening, resulted in the development of several promising soybean lines for release as cultivars or use as parents in breeding programs. These candidate lines include 91–38, 52–82B, SSR51–70, and 51–23. Line 91–38 achieved an average yield of 2,802.5 kg ha^-1^, which is 360.2 kg ha^-1^ higher than W04–1002, the Sclerotinia stem rot resistant parent, and a mean DSI value of 11.4 across all field years evaluated. Line 91–38, which possessed the novel resistance-associated marker region on chromosome 16, also had one of the lowest disease severity rankings in both field and greenhouse trials compared to the susceptible check, Dwight, and other commercial lines in 2016. Additionally, line 52–82B had one of the best yields, a 3-year mean of 3,547.1 kg ha^-1^, and a low DSI mean of 27.5. Line SSR51–70 consistently exhibited among the lowest disease scores for all years in both field (mean DSI of 10.7) and greenhouse studies. With a 3-year mean yield of 2,972.5 and DSI of 26.2, line 51–23 also exhibits promising yield potential and a high level of Sclerotinia stem rot resistance. All lines yielded on average between 2,700 and 3,600 kg ha^-1^ and were consistently near or above the yearly state averages for 2014 (2,953.03 kg ha^-1^), 2015 (3,322.15 kg ha^-1^), and 2016 (3,691.27 kg ha^-1^) (National Agricultural Statistics Services, 2014–2016). Overall, the yield performance and elevated disease resistance of these four lines provides strong evidence for their candidacy in future Sclerotinia stem rot resistance breeding programs.

Additionally, lines 91–38, 52–82B, and 51–23 exhibit reduced lodging phenotypes, another highly desirable agronomic trait. Lodging was correlated with lower yield in 2014 (-0.56, *P* < 0.0001), but it was not associated with disease severity. This is not surprising as previous findings have associated lower lignin content, a component of structural tissues in vascular plants, with a high level of resistance to Sclerotinia stem rot (Peltier et al., 2009). It has also been suggested that lower lignin content can act as a biological marker for Sclerotinia stem rot resistance; however, decreased lignin levels are likely related to increased lodging (Boland and Hall, 1987). The negative correlation between yield and lodging has been observed in other studies (Jin et al., 2010; Recker et al., 2014). Others have also found positive correlations between Sclerotinia stem rot DSI and lodging (Kim et al., 1999). Line 91–38, however, is an example of a line exhibiting both disease resistance in multiple environments and minimal lodging characteristics. While lodging is typically associated with increased Sclerotinia stem rot resistance and reduced yield, the candidate lines presented here consistently exhibit low lodging scores with near or above average yields and strong Sclerotinia stem rot resistance.

Furthermore, lines 91–38 and 51–23 could be considered as food-grade soybean releases, possessing a yellow hilum and high protein levels. In fact, most lines developed here possessed average protein contents above 36% and oil contents that were near 20% (both on a 13% moisture basis), which is above average for soybeans grown in Wisconsin (Miller-Garvin and Naeve, 2016). Line 91–38 also had the best balance of high protein and oil content of the four lines indicated above. Considering the high level of Sclerotinia stem rot resistance, high protein and oil content, and yellow hilum trait, this line has been designated as a candidate for release as a non-GMO, food-grade soybean variety. It will be available as the variety Dane through agreements with Wisconsin Foundation Seeds^2^. Other lines reported here are available for breeding purposes through an agreement with the Wisconsin Alumni Research Foundation (WARF).

The work presented here demonstrates that genetic gain can be made for Sclerotinia stem rot resistance without sacrificing agronomic qualities in soybean when a holistic approach of marker-assisted selection, greenhouse screening, and field disease nursery screening are used together. Furthermore, we have validated a proof of concept that genetic gain for physiological Sclerotinia stem rot resistance can be achieved, independent of klendusity, through selection in a controlled greenhouse environment using petiole inoculations. We were able to identify several soybean lines that have excellent potential as parents in a breeding program or as varieties themselves, as evidenced by the planned release of 91–38. In addition, crosses have been performed using lines 51–23, SSR51–70, and 52–82B to identify new germplasm lines with even greater Sclerotinia stem rot resistance through combining sources of resistance while maintaining yield potential.

## Author Contributions

BD, CRG, DLS, and MK developed the study and designed the experimental trials. CG, JW, and MM implemented the evaluations. DLS, JW, and MM conducted the data analyses. MM, JW, and AR prepared tables, figures, and wrote the manuscript. BD, MK, and AR conducted the QTL evaluations. All authors reviewed the manuscript. MK and DLS are the corresponding authors on this manuscript and the PIs of the project under which this study was carried out.

## Conflict of Interest Statement

The authors declare that the research was conducted in the absence of any commercial or financial relationships that could be construed as a potential conflict of interest.

## Footnotes

1 http://soybase.org

2 https://wisconsinfoundationseeds.wisc.edu
