# Supplementary material for: Development and Evaluation of Glycine max Germplasm Lines with Quantitative Resistance to Sclerotinia sclerotiorum
Source: Front Plant Sci. 2017 Aug 31;8:1495. doi: 10.3389/fpls.2017.01495 (PMC5584390; doi:10.3389/fpls.2017.01495)
Supplement: Supplementary file 1 [file Table_1.pdf]

Supplementary Table S1. Mean lodging scores for breeding lines in 2014

Variety	Mean Lodging Score[z]	Rank Estimate[y]
91-145	3.4	62.4 a
51-23	3.0	56.0 ab
91-103	3.0	56.0 ab
SSR51-70	3.0	53.8 abc
W04-1002	3.0	51.6 abcd
SSR81-62	2.8	50.6 abc
41-39	2.6	45.2 bcd
52-14	2.2	35.6 cdef
SSR81-107	2.2	34.4 de
91-44	2.0	29.0 e
52-82B	1.6	20.6 ef
Dwight	1.6	20.6 ef
52-11	1.5	18.5 efg
91-38	1.4	16.4 fg
AxN-1-55	1.0	8.0 g

[z]Soybean lodging was scored on a 1-5 scale: 1= upright. 2 = 25-degree lean. 3 = 45-degree lean. 4 = more than 45-degree lean and 5 = laying on the ground. This scale represents the approximate average rating for the whole plot.

[y]Rank estimates were generated to compare categorical lodging scores. Values followed by the same letter are not significantly different based on Fisher's Least Significant Difference (LSD; $\alpha=0.05$).
