# Supplementary material for: Development and Evaluation of Glycine max Germplasm Lines with Quantitative Resistance to Sclerotinia sclerotiorum
Source: Front Plant Sci. 2017 Aug 31;8:1495. doi: 10.3389/fpls.2017.01495 (PMC5584390; doi:10.3389/fpls.2017.01495)
Supplement: Supplementary file 2 [file Table_2.pdf]

Supplementary Table S2. Mean lodging scores for breeding lines in 2016

Variety	Mean Lodging Score[a]	Rank Estimate[b]
91-145	3.4	48.3 a
SSR51-70	3.2	46.2 a
91-44	2.4	36.5 ab
W04-1002	2.0	29.4 bc
51-23	2.0	33.0 b
Dwight	1.6	26.8 bc
52-82B	1.3	21.3 bcd
52-11	1.2	17.9 cd
AxN-1-55	1.0	14.5 d
91-38	1.0	14.5 d

[a]Soybean lodging was scored on a 1-5 scale: 1= upright. 2 = 25-degree lean. 3 = 45-degree lean. 4 = more than 45-degree lean and 5 = laying on the ground. This scale represents the approximate average rating for the whole plot.

[b]Rank estimates were generated to compare categorical lodging scores. Values followed by the same letter are not significantly different based on Fisher's Least Significant Difference (LSD; α=0.05).
