# Supplementary material for: Development and Evaluation of Glycine max Germplasm Lines with Quantitative Resistance to Sclerotinia sclerotiorum
Source: Front Plant Sci. 2017 Aug 31;8:1495. doi: 10.3389/fpls.2017.01495 (PMC5584390; doi:10.3389/fpls.2017.01495)
Supplement: Supplementary file 3 [file Table_3.pdf]

Supplementary Table S3. Total protein and total oil content of grain from soybean breeding lines harvested in 2015 calibrated to 13% moisture

Breeding Line	Protein (%)[z]	Oil (%)[z]
91-103	38.4 a	18.2 h
W04-1002	38.1 ab	17.8 i
91-38	37.8 abc	19.0 bc
SSR51-70	37.5 bd	18.6 df
52-14	37.3 cde	18.7 cde
91-44	37.1 de	19.6 a
52-11	37.1 de	18.5 efgh
91-145	37.1 de	18.2 gh
52-82B	37.0 df	18.5 dg
AxN-1-55	37.0 df	18.4 efgh
SSR81-62	36.8 ef	18.2 h
SSR81-107	36.4 fg	19.2 b
Dwight	35.7 gh	18.8 cd
51-23	35.5 h	18.3 fgh
41-39	35.3 h	19.3 ab

[z]Means followed by the same letter are not significantly different based on Fisher's Least Significant Difference (LSD; α=0.05).
