# Supplementary material for: Development and Evaluation of Glycine max Germplasm Lines with Quantitative Resistance to Sclerotinia sclerotiorum
Source: Front Plant Sci. 2017 Aug 31;8:1495. doi: 10.3389/fpls.2017.01495 (PMC5584390; doi:10.3389/fpls.2017.01495)
Supplement: Supplementary file 4 [file Table_4.pdf]

Supplementary Table S4. Total protein and total oil content of grain from soybean breeding lines harvested in 2016 calibrated to 13% moisture.

Breeding Line	Protein (%)[a]	Oil (%)[a]
W04-1002	39.4 a	17.9 c
SSR51-70	39.2 ab	18.6 b
91-44	38.7 ac	19.2 a
91-38	38.6 bc	19.2 a
52-82B	38.5 bc	18.1 c
52-11	38.5 c	18.4 b
AxN-1-55	38.3 c	18.4 b
91-145	37.6 d	18.7 b
Dwight	37.6 d	18.5 b
51-23	37.3 d	18.5 b

[a]Means followed by the same letter are not significantly different based on Fisher's Least Significant Difference (LSD; α=0.05).
